# Use of Custom-Designed Additive-Manufactured Acetabular Components for Reconstruction of Paprosky Type III Acetabular Defects in Revision Hip Arthroplasty: A Single-Center Case Series

**DOI:** 10.3390/jcm15093416

**Published:** 2026-04-29

**Authors:** Alexey A. Belokobylov, Valery D. Serikbayev, Konstantin A. Petrovsky, Evgeniy A. Novik, Bagdat N. Azamatov, Yersultan E. Alzhanov, Darkhan. B. Sultanov, Lyudmila V. Spichag

**Affiliations:** 1 National Scientific Center of Traumatology and Orthopedics, Astana 010000, Kazakhstan; belokobylov_a@nscto.kz (A.A.B.); serikbayev_v@nscto.kz (V.D.S.); petrovsky_k@nscto.kz (K.A.P.); novik_e@nscto.kz (E.A.N.); alzhanov_e@nscto.kz (Y.E.A.); spichag_l@nscto.kz (L.V.S.); 2 Non-Profit Joint-Stock Company, D. Serikbayev East Kazakhstan Technical University, Astana 010000, Kazakhstan; bazamatov@edu.ektu.kz

**Keywords:** revision hip arthroplasty, acetabular bone defect, Paprosky classification, three-dimensional printing (3D printing), custom-made implant, pelvic discontinuity

## Abstract

**Background/Objectives:** To evaluate the short-term clinical and radiological outcomes of using custom-designed additive-manufactured acetabular components (CDAMACs) in revision total hip arthroplasty for patients with Paprosky type IIIA-IIIB acetabular defects. **Materials and Methods:** A retrospective single-center case series was conducted. Between 2020 and 2025, 19 patients with massive Paprosky type IIIA-IIIB acetabular defects underwent revision hip arthroplasty with CDAMACs. Preoperative planning was based on multislice computed tomography data, followed by 3D modeling and implant design. Perioperative parameters, functional outcomes (Harris Hip Score [HHS], WOMAC, Visual Analog Scale [VAS] for pain), and radiographic parameters (restoration of the center of rotation, component stability) were assessed. Minimum follow-up was 12 mo. **Results:** The mean operative time was 155 ± 24 min, and the mean blood loss was 718 ± 288 mL. At 12 mo, significant functional improvements were observed: the mean HHS increased from 37.5 ± 5.2 to 74.5 ± 8.6 points, WOMAC decreased from 74.5 ± 9.2 to 40.3 ± 7.6 points, and VAS decreased from 7.6 ± 1.0 to 2.8 ± 0.7 points (*p* < 0.001 for all). Restoration of the hip center of rotation was determined. Minimum follow-up was 12 mo. No component migration or progressive radiolucent lines were observed. Complications occurred in two patients (10.5%), with only one case directly related to the acetabular component. **Conclusions:** The use of CDAMACs in revision hip arthroplasty for severe Paprosky type IIIA-IIIB acetabular defects is associated with satisfactory short-term clinical, functional, and radiological outcomes. This technique enables restoration of the center of rotation and provides stable component fixation in complex anatomical conditions.

## 1. Introduction

Total hip arthroplasty (THA) is one of the most successful orthopedic procedures, providing significant improvement in patients’ quality of life [1]. However, due to increased life expectancy and expanded indications for primary arthroplasty, a steady rise in revision procedures is observed worldwide; for example, in the United Kingdom, the number of revision hip arthroplasties increased from approximately 6000 in 2014 to over 8000 in 2023 [2]. The management of patients with massive acetabular bone defects, particularly Paprosky types IIIA and IIIB, remains one of the most challenging tasks in revision arthroplasty. These defects are characterized by substantial bone loss (>30%), disruption of the pelvic supporting columns, and instability of the pelvic ring, rendering standard reconstruction methods largely ineffective [3,4].

This trend is also evident in the Republic of Kazakhstan, where, according to the Ministry of Health, the number of revision procedures has more than doubled from 2019 to 2023, underscoring the growing need for effective reconstructive technologies. The main causes leading to massive acetabular defects are aseptic loosening, periprosthetic joint infection, and wear particle-induced osteolysis [5,6].

Traditional reconstruction methods, such as bone grafting, antiprotrusio cages, and cage systems, often fail to restore the anatomical center of rotation and provide stable long-term fixation in Paprosky type III defects, resulting in high re-revision rates [7]. Consequently, additive manufacturing technologies have garnered increasing attention. Custom-designed additive-manufactured acetabular components enable the creation of implants that precisely match the unique anatomy of a patient’s defect, providing multi-point fixation and restoration of joint biomechanics [8,9].

Data accumulated to date, including initial systematic reviews, demonstrate encouraging short- to mid-term outcomes with custom-designed additive-manufactured acetabular components (CDAMACs). Early implant survival rates reach 97.7%, and functional scores (Harris Hip Score) improve significantly [10,11]. Recent systematic reviews have further corroborated these findings, reporting implant survival rates of 95.5% at a mean follow-up of 3.8 years and significant functional improvement in patients with Paprosky type III defects [12,13]. Baauw et al. [14] first reported the use of custom triflange implants for Paprosky type III defects with good early fixation. Aprato et al. [15] confirmed these findings in a series of 18 patients, showing significant pain relief and functional recovery. Gruber et al. [16] and Froschen et al. [17] provided mid-term evidence of stable osseointegration and implant survival exceeding 90% at 5 years. Von Hertzberg-Boelch et al. [18] emphasized the importance of accurate 3D planning for achieving rotational center restoration. Studies with longer follow-up (up to 7 years) confirm stable fixation and osseointegration in most patients [19].

However, despite promising results published by international centers, data on the application of this technology in the Republic of Kazakhstan and other post-Soviet countries remain extremely limited. Existing studies often include heterogeneous patient groups and have short follow-up periods [10]. This justifies the need to accumulate local clinical experience and analyze outcomes of custom-made 3D implants within a specific healthcare system.

Objective: To evaluate the short-term clinical and radiological outcomes of custom-made 3D-printed acetabular components in revision hip arthroplasty for patients with Paprosky type IIIA-IIIB acetabular defects.

## 2. Materials and Methods

### 2.1. Study Design

This study is a retrospective single-center case series. It was conducted at the National Scientific Center of Traumatology and Orthopedics (Astana, Republic of Kazakhstan) from January 2020 to December 2025. All surgical procedures were performed based on clinical indications as part of standard medical care. The minimum postoperative follow-up was 12 mo.

This study report was prepared in accordance with the STROBE (Strengthening the Reporting of Observational Studies in Epidemiology) guidelines for observational studies [20].

### 2.2. Inclusion and Exclusion Criteria

Patient selection was based on the following criteria, defined according to the Paprosky classification of acetabular bone defects [3] and the International Consensus Meeting (ICM) criteria for periprosthetic joint infection diagnosis [21].

Inclusion Criteria:

1. Presence of a Paprosky type IIIA or IIIB acetabular bone defect, confirmed by preoperative imaging and intraoperatively.
2. Revision hip arthroplasty using a CDAMAC.
3. Completed clinical and radiological follow-up of at least 12 months postoperatively.
4. Signed informed consent to participate in the study.

Exclusion Criteria:

1. Active periprosthetic joint infection at the time of revision surgery (diagnosed according to the 2018 ICM criteria).
2. Neoplastic involvement of the pelvic bones (primary or metastatic) in the planned reconstruction area.
3. Severe decompensated comorbidities (ASA class IV or higher) precluding safe surgical intervention and postoperative rehabilitation.
4. Lack of signed informed consent or patient withdrawal from the study at any stage.

### 2.3. Ethical Considerations

This study was conducted in accordance with the principles of the Declaration of Helsinki (2013 revision) [22], the Code of the Republic of Kazakhstan “On Public Health and the Healthcare System” (No. 360-VI, dated 7 January 2020), and the Order of the Minister of Health of the Republic of Kazakhstan No. ҚP ДCM-310/2020 dated 21 December 2020.

This study protocol was approved by the local Ethics Committee of the National Scientific Center of Traumatology and Orthopedics (Astana, Republic of Kazakhstan) (Protocol No. 4/1 dated 25 December 2024).

Due to the retrospective nature of this study and the use of only de-identified data, the local Ethics Committee waived the requirement for obtaining separate informed consent from patients. Confidentiality of personal data is ensured in accordance with the current legislation of the Republic of Kazakhstan on personal data protection.

### 2.4. Demographic and Clinical Patient Characteristics

This study included 19 patients: 10 men and 9 women. The mean age at surgery was 53.6 ± 11.0 years (range: 39–72 years). Left-sided involvement was noted in 11 (57.9%) patients, right-sided in 8 (42.1%). The mean number of previous hip surgeries was 3.0 ± 2.1 (median 2; range 1–9). The mean interval from primary arthroplasty to revision surgery using the 3D component was 13.7 ± 6.2 years (median 12; range 4–25 years). A history of periprosthetic infection was recorded in 8 (42.1%) patients. According to the Paprosky classification, 6 (31.6%) patients had a type IIIA defect and 13 (68.4%) had a type IIIB defect. Pelvic discontinuity was present in 16/19 patients (84.2%). According to the original Paprosky classification, type IIIA defects show 2–3 cm of proximal migration of the hip center, while type IIIB defects show >3 cm of proximal migration with associated pelvic discontinuity or ischial lysis [3]. Detailed demographic and clinical data are presented in Table 1.

### 2.5. Preoperative Assessment and Decision-Making Algorithm

All patients underwent preoperative multislice computed tomography (MSCT) of the pelvis using a Siemens Somatom Definition AS 40 scanner (Siemens Healthineers, Erlangen, Germany) with a bone reconstruction algorithm (Bone+) and 0.6 mm slice thickness. Imaging was performed without intravenous contrast. The acquired DICOM images were exported for further processing and used for detailed assessment of:

- Type and extent of the acetabular defect according to the Paprosky classification [3];
- Bone loss volume and quality of remaining bone (ilium, ischium, and pubis);
- Integrity of the anterior and posterior acetabular columns;
- Presence of signs of pelvic discontinuity [5].

The decision-making algorithm for using a custom-made 3D-printed acetabular component was standardized and included the following steps:

1. Analysis of clinical and radiological data, including defect classification according to Paprosky and determination of the feasibility of using standard revision constructs (e.g., antiprotrusio cages, metal augments, bone grafting).
2. Consensus decision on the impossibility of achieving stable primary fixation and restoring the anatomical center of rotation using traditional methods due to the massive bone defect (type IIIA-IIIB) or disruption of pelvic support structures.
3. Transfer of DICOM data to an engineering team for custom implant design.
4. Final approval of the treatment plan by a multidisciplinary team, including operating orthopedic surgeons and biomedical engineers.

### 2.6. 3D Planning and Fabrication of the Custom Acetabular Component

Following clinical decision approval, de-identified DICOM datasets were transferred to an engineering company specializing in additive manufacturing (LOGIX Medical Systems LLC, Novosibirsk, Russia) for the design and fabrication of the custom acetabular component. Implant design was based on MSCT data, incorporating the results of preoperative clinical-spatial defect assessment, including determination of the optimal center of rotation position, extent of bone resection, and planning of screw trajectories. To verify the design and rehearse the surgical stage, a physical nylon model of the pelvis was fabricated using 3D printing, allowing the surgical team to refine implant positioning and finalize its design collaboratively.

Custom acetabular components were manufactured using direct metal laser sintering (DMLS) from Ti6Al4V titanium alloy (LPW Technology Ltd., Lydney, UK), conforming to ASTM F136 (UNS R56401) standard [8]. The Ti6Al4V powder was gas-atomized (EOS Titanium Ti64 Grade 23) with a particle size distribution of D10 = 15 µm, D50 = 28 µm, D90 = 45 µm, conforming to ASTM F136. Additive manufacturing was performed using an EOS M 290 direct metal laser sintering system (EOS GmbH, Krailling, Germany) with the following parameters: laser power 400 W, scanning speed 1000 mm/s, layer thickness 20 μm, and hatching distance 100 μm, resulting in an energy density of approximately 60 J/mm^3^. These parameters are standard for medical-grade titanium implants and ensure high density (>99.5%) and precise reproduction of fine features.

The porous surface was fabricated directly by DMLS without additional coating. Pore sizes ranged from 300 to 700 μm (average 500 μm), and porosity was approximately 60–70%, fully interconnected, as designed in the CAD model and verified by micro-CT. The porous layer thickness was 1.5 mm on all bone-contacting surfaces [23,24].

The implant design included three flanges for fixation to the remaining ilium, ischium, and pubis, providing multi-point load distribution and enhancing primary mechanical stability. After additive manufacturing, multi-axis machining of the bearing surfaces and screw holes was performed to achieve precise positioning and fit to the individual anatomy.

**Figure 1:**
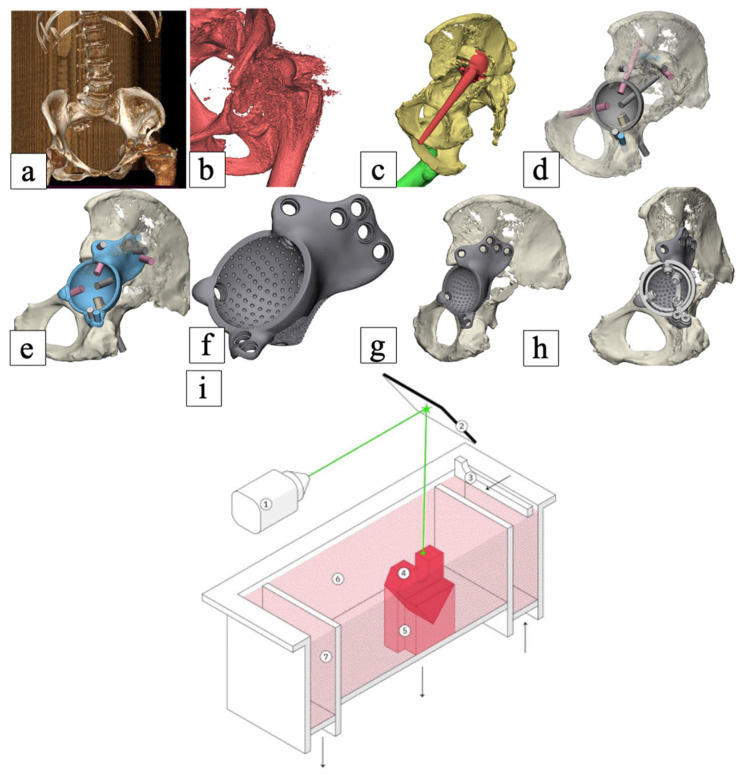
Algorithm for creation of custom-designed additive-manufactured implant. (**a**) CT scan of the pelvis (Siemens Somatom Definition AS 40); (**b**) STL model creation from CT data; (**c**) processing of the obtained model; segmentation by elements; (**d**) defect volume assessment; positioning of the cup and screws; (**e**) construction of the basic implant shape; (**f**) detailing of the implant design; (**g**) clinical discussion of the obtained model with orthopedic surgeons; (**h**) design of additional elements; (**i**) schematic diagram of the direct metal laser sintering (DMLS) process: 1—laser source; 2—XY scanning mirror; 3—piston/elevator; 4—built part (3D model); 5—support structures; 6—metal powder bed; 7—excess powder reservoir.

The detailed sequence of preoperative 3D planning and custom implant creation is presented in Figure 1 (from CT acquisition to implant manufacturing using DMLS).

### 2.7. Surgical Technique

All surgical procedures were performed through a single anterolateral approach (Watson–Jones) with the patient in the lateral decubitus position. After exposing the acetabular region and removing previously implanted unstable components, thorough debridement of the bony bed, excision of fibrous membranes and lavage were performed. A preoperatively fabricated sterile additive-manufactured pelvis model (nylon or sterilizable photopolymer) was used to verify the correspondence between the defect area and the planned implant position, allowing refinement of flange orientation and screw trajectories. The custom triflange acetabular component was positioned according to the preoperative plan to restore the anatomical center of rotation. Implant fixation was achieved using 6.5 mm cortical screws placed through the flanges into the remaining ilium, ischium, and pubis. A minimum of three screws were used, with the number increased to 5–6, as needed, to achieve stable multi-point fixation, particularly in cases of pelvic discontinuity [8]. The custom triflange component was designed to intimately fill the cavitary and segmental bone defect, occupying the void left after removal of the failed implant. The porous undersurface of the component directly contacts the remaining host bone in the ilium, ischium, and pubis, while the three flanges bridge the defect and provide cortical fixation. Any residual dead space between the implant and the bone was filled with morselized autograft or allograft bone chips to promote osseointegration.

**Figure 2:**
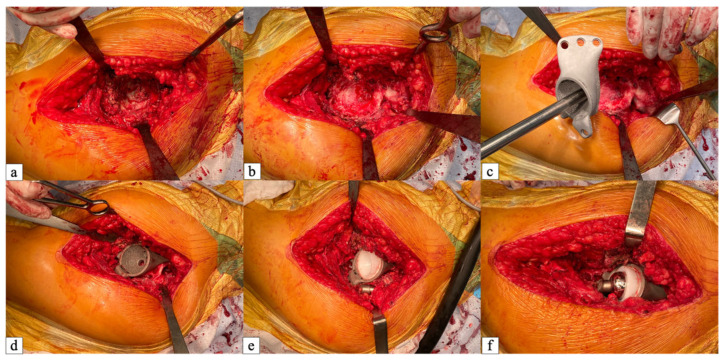
Intraoperative photographs of the surgical sequence using a custom-designed additive-manufactured acetabular component (CDAMAC). (**a**) Massive acetabular bone defect (Paprosky type IIIB) before preparation; (**b**) same defect after debridement and reaming, showing the remaining bone stock; (**c**) trial positioning of the custom triflange component; (**d**) definitive fixation of the CDAMAC with cortical screws engaged in the ilium, ischium, and pubis; (**e**) cementation of the highly crosslinked polyethylene liner; (**f**) final reduction of the femoral head component, with stable hip range of motion.

After component insertion and fixation, primary stability was assessed. A conventional highly crosslinked polyethylene liner (non-retentive, non-raised) was cemented into the component using antibiotic-loaded bone cement (1 g of vancomycin per 40 g of cement). Femoral head sizes varied depending on intraoperative assessment and available implants: 28 mm heads were used in 12 patients and 32 mm heads in the remaining 7 patients. Dual mobility cups or large heads (>36 mm) were not used because the constrained acetabular bone stock required stable cementation of the liner within the custom triflange component. Stability of the entire construct, absence of impingement, range of motion, and restoration of soft tissue tension were evaluated. The wound was closed in layers over a suction drain. Intraoperative steps of implant placement are shown in Figure 2.

### 2.8. Clinical and Radiological Outcome Assessment

Clinical and functional assessments were performed preoperatively and at 3, 6, and 12 mo post-surgery. The following validated instruments were used:

- Harris Hip Score (HHS)—to assess overall functional status, pain, and range of motion [25];
- WOMAC (Western Ontario and McMaster Universities Osteoarthritis Index)—to assess pain, stiffness, and functional limitations related to the hip joint [26];
- Visual Analog Scale (VAS)—to quantify pain intensity (0 to 10 points) [27].

Radiological evaluation was performed for all patients in the early postoperative period (first 3–5 days) and then at 3, 6, and 12 months. Assessment was based on standard anteroposterior pelvic radiographs, including both hip joints. If component instability or migration was suspected, follow-up MSCT was performed to verify implant position and assess bone integration.

The following radiological parameters were analyzed:

1. Restoration of the center of rotation was defined as the distance from the prosthetic femoral head center to the interteardrop line in the vertical plane and to bony landmarks (e.g., obturator foramen) in the horizontal plane. Center of rotation displacement of less than 3 mm in any direction was considered anatomical restoration [13].
2. Fixation stability was assessed by the presence or absence of component migration (change in inclination angle or position by more than 3 mm or 3° compared to postoperative images) [28].
3. Radiolucent lines were evaluated in the DeLee and Charnley zones for the acetabular component [29] and the Gruen zones for the femoral component [30]. Progressive lines > 2 mm wide in two or more zones were considered a sign of possible loosening.
4. Osseointegration was assessed according to the criteria of Moore et al. (presence of trabecular remodeling, absence of radiolucent lines, presence of stress shielding in supporting zones) [31].

### 2.9. Statistical Analysis

Statistical analysis was performed using IBM SPSS Statistics version 26.0 (IBM Corp., Armonk, NY, USA). Descriptive statistics were used for quantitative variables. Normality of distribution was assessed using the Shapiro–Wilk test [32]. All analyzed quantitative parameters (HHS, WOMAC, VAS) followed a normal distribution and are presented as mean ± standard deviation (M ± SD). Categorical variables are described as absolute (n) and relative (%) frequencies.

To compare functional outcomes (HHS, WOMAC, VAS) over time (preoperative, 3, 6, and 12 mo), repeated measures ANOVA was used, followed by post-hoc pairwise comparisons with Bonferroni correction for multiple testing [33]. This method allows assessment of both the overall effect of time and the significance of differences between individual time points while minimizing type I errors.

Differences were considered statistically significant at a two-tailed *p* < 0.05. A post-hoc power analysis was not conducted because the primary objective of this case series was descriptive. The small sample size limits generalizability, and the results should be considered hypothesis-generating, requiring confirmation in larger prospective cohorts [34]. Therefore, the obtained results should be considered preliminary and require confirmation in studies with larger patient cohorts.

## 3. Results

### 3.1. Perioperative Parameters

All 19 patients underwent elective revision hip arthroplasty using custom-made 3D-printed acetabular components. The mean operative time was 155 ± 24 min (range 120–210 min). The mean intraoperative blood loss was 718 ± 288 mL (range 300–1500 mL). Blood product transfusion (packed red blood cells or fresh frozen plasma) was required in four cases (22%).

The mean postoperative hospital stay was 14.4 ± 6.6 days (median 12 days; range 7–29 days). No perioperative deaths occurred.

### 3.2. Functional Outcomes

Preoperatively, all patients experienced severe pain and functional limitations. The mean HHS was 37.5 ± 5.2 (95% CI: 35.0–40.0), the mean WOMAC was 74.5 ± 9.2 (95% CI: 70.1–78.9), and the mean VAS pain score was 7.6 ± 1.0 (95% CI: 7.1–8.1).

At 3, 6, and 12 mo postoperatively, statistically significant improvements were observed across all three scales (*p* < 0.001 for all time points compared to baseline). The dynamics of the scores are presented in Table 2.

At 12 mo, the mean HHS reached 74.5 ± 8.6 (95% CI: 70.3–78.7), WOMAC decreased to 40.3 ± 7.6 (95% CI: 36.6–44.0), and VAS decreased to 2.8 ± 0.7 (95% CI: 2.5–3.1). Differences from preoperative levels were statistically significant (*p* < 0.001, paired t-test with Bonferroni correction for multiple comparisons).

### 3.3. Radiological Outcomes

Radiological evaluation was performed for all patients in the early postoperative period (first 3–5 days) and at 3, 6, and 12 mo postoperatively using standard anteroposterior pelvic radiographs. Assessment followed the recommendations of DeLee and Charnley for the acetabular component [29] and Gruen for the femoral component [30].

**Figure 3:**
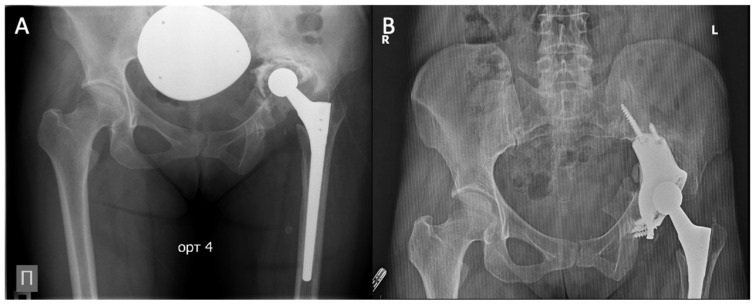
Radiographs of a 52-year-old female patient with a Paprosky type IIIB defect: (**A**) Preoperative; (**B**) 12 months after reconstruction with a custom-designed additive-manufactured acetabular component.

The mean vertical displacement of the hip center was 4.5 mm (range 1–12 mm). Restoration to within 3 mm of the anatomical position was achieved in 12 of 19 patients (63.2%). In the remaining seven patients, residual displacement ranged from 4 to 12 mm, reflecting the severity of bone defects and pelvic discontinuity. An example of successful reconstruction with minimal displacement (2 mm) is shown in Figure 3. No signs of acetabular component migration (position change >3 mm or inclination change >3°) or progressive radiolucent lines >2 mm wide in the DeLee–Charnley zones were observed during the follow-up period in any patient.

In two patients (10.5%), deviation of the center of rotation from the anatomical position (>3 mm displacement) was observed, attributed to the initial severity of the bone defect (both cases were Paprosky IIIB with pelvic discontinuity). In one of these cases, component instability was diagnosed at 8 mo postoperatively, necessitating repeat revision surgery (detailed in the “Complications” section).

### 3.4. Complications and Adverse Events

During the 12-month follow-up period, complications were recorded in 2 of 19 patients (10.5%). Complications were classified as directly related or not directly related to the acetabular component.

Complications directly related to the acetabular component: In one patient (5.3%), a 59-year-old woman with a Paprosky type IIIB defect and pelvic discontinuity, aseptic loosening of the custom 3D implant was diagnosed 8 mo postoperatively. Clinically, this manifested as load-dependent pain and decreased functional scores (HHS decreased from 72 to 58). Radiologically, component migration >5 mm and progressive radiolucent lines in DeLee zones II and III were observed. The patient underwent repeat revision surgery with replacement of the acetabular component with a newly designed implant. Positive dynamics were noted after reoperation; at 12 mo post-revision, HHS was 81, with no signs of instability.

Complications not directly related to the acetabular component: In one patient (5.3%), a 25-year-old man with post-traumatic pelvic deformity and pelvic discontinuity, deep periprosthetic joint infection developed after staged surgical treatment aimed at correcting limb length discrepancy (diagnosed according to 2018 ICM criteria [35]). Surgical debridement with an exchange of mobile components and subsequent long-term antibiotic therapy were performed. The infection was controlled, and the implant was retained. This case was not directly related to the placement of the custom 3D-printed acetabular component.

No cases of prosthetic dislocation, acetabular component fracture, thromboembolic events, or deaths were recorded during the entire follow-up period.

## 4. Discussion

Reconstruction of massive acetabular defects in revision hip arthroplasty remains one of the most challenging problems in contemporary orthopedics. Significant bone loss, distortion of anatomical landmarks, and, in some cases, pelvic discontinuity substantially limit the potential of standard reconstructive techniques and increase the risk of unstable acetabular component fixation [3,4,5].

In the present study, the use of CDAMACs in patients with Paprosky type IIIA-IIIB defects was associated with satisfactory short-term clinical, functional, and radiological outcomes. At 12 mo postoperatively, statistically significant improvements were observed in HHS (from 37.5 to 74.5 points), WOMAC (from 74.5 to 40.3 points), and VAS (from 7.6 to 2.8 points) (*p* < 0.001). Anatomical restoration of the center of rotation (<3 mm displacement) was achieved in 63.2% of patients (12/19), with a mean vertical displacement of 4.5 mm (range 1–12 mm). No evidence of migration or progressive radiolucent lines was observed in the majority of patients. These findings align with results from previously published case series [8,13,19].

Comparison with the Literature. Functional outcomes in our study are consistent with those reported by other authors. Goriainov et al. [8] reported an increase in HHS from 38 to 77 points at 12 months in patients with Paprosky III defects. Martinez et al. [13] observed HHS improvement from 41.8 to 83.9 points at a mean follow-up of 3.8 years. In a systematic review by Almeida et al. [12], a subgroup analysis of patients with Paprosky type III defects revealed a mean postoperative HHS of 86.7 and an implant survival rate of 95.5% at a mean follow-up of 3.8 years. Our 12-month HHS of 74.5 is lower than these longer-term results, which is expected given the shorter follow-up and the severity of our cohort (68.4% Paprosky IIIB, 84.2% with pelvic discontinuity). The implant survival of 94.7% in our series compares favorably with the 95.5% reported by Almeida et al., indicating reliable early fixation even in the most complex anatomical situations.

Radiological outcomes also correspond to international data. Restoration of the anatomical center of rotation to within 3 mm was achieved in 63.2% of our patients, which is comparable to other series of Paprosky III defects with pelvic discontinuity [14,15]. The absence of progressive radiolucent lines and migration in most of our patients indicates stable primary fixation and potential for osseointegration, supported by the porous implant structure (pore size 300–700 μm, porosity 60–70%) consistent with optimal parameters for bone ingrowth [18,19].

Complications. The complication rate in our series (10.5%) falls within the range reported in the literature for revisions using custom implants. Goriainov et al. [8] reported complications in 16% of patients, while Martinez et al. [13] reported 17.4% (including two cases of periprosthetic infection). In a large meta-analysis, Almeida et al. [12] found a re-revision rate of 4.48% for any reason, which is comparable to our single aseptic loosening requiring re-revision (5.3%). Importantly, the second complication (periprosthetic infection) in our series occurred after an additional staged procedure and was not directly related to the acetabular component. The absence of dislocations and mechanical failures confirms the reliability of multi-point fixation and the accuracy of preoperative planning.

The absence of prosthetic dislocation in our series (0/19) warrants specific comment. Despite the use of conventional non-retentive polyethylene liners and mixed femoral head sizes (28 mm in 12 patients, 32 mm in 7 patients), no dislocations occurred during the 12-month follow-up. We attribute this to three main factors: (1) accurate restoration of the anatomical center of rotation (achieved in 63.2% of patients), which optimizes soft tissue tension; (2) stable multi-point fixation of the custom triflange component, which prevents abnormal component motion and preserves native hip biomechanics; and (3) meticulous intraoperative assessment of range of motion and impingement, with adjustment of component orientation as needed. While dual mobility cups have been advocated for to reduce dislocation risk in revision arthroplasty, their use was not feasible in our cohort due to the large acetabular defects that required custom-designed fixed-shell components. The cementation of a conventional liner into the custom shell proved reliable in these complex cases, with no dislocations observed at short-term follow-up.

Advantages and Limitations. The key advantage of custom-designed additive-manufactured implants is their ability to precisely adapt to complex defect anatomy, enabling restoration of joint biomechanics and providing primary stability even in the presence of pelvic discontinuity [8,36]. However, the method has limitations, including high cost, long production times, and the need for close collaboration between surgeons and engineers, which may limit its widespread application, particularly in healthcare systems with constrained funding [36]. Furthermore, regulatory approval requirements (e.g., from the Ministry of Health of the Republic of Kazakhstan, as well as conformity to standards (e.g., GOST ISO 13485-2017 [37]) add complexity. Our study also has several methodological limitations: its retrospective design, small sample size (n = 19), lack of a control group, relatively short follow-up (12 mo), and the fact that we did not investigate the influence of DMLS process variables (e.g., laser power, scanning speed, hatching distance) on the mechanical and biological performance of the fabricated implants. These preclude definitive conclusions regarding long-term implant survival and necessitate confirmation in prospective studies with larger cohorts and longer follow-up.

Clinical Significance. Despite these limitations, the data obtained suggest that custom-made 3D-printed acetabular components are a viable option for treating patients with the most severe acetabular defects (Paprosky IIIA-IIIB) where standard reconstruction methods are ineffective. Meticulous preoperative planning, a multidisciplinary approach, and adherence to surgical technique can yield good short-term results with an acceptable complication rate. Accumulating experience and further research will help refine indications and optimize the application of this technology.

## 5. Conclusions

The use of custom-designed, additive-manufactured acetabular components in patients with Paprosky type IIIA-IIIB acetabular defects demonstrated satisfactory short-term clinical, functional, and radiological outcomes. At 12 mo postoperatively, statistically significant improvements were observed in HHS (from 37.5 to 74.5 points), WOMAC (from 74.5 to 40.3 points), and VAS (from 7.6 to 2.8 points) (*p* < 0.001). Anatomical restoration of the center of rotation (displacement < 3 mm) was achieved in 12/19 patients (63.2%), with a mean vertical displacement of 4.5 mm. No signs of migration or progressive radiolucent lines were observed, confirming stable early fixation.

The use of custom triflange constructs provided reliable multi-point fixation even in the setting of severe bone loss and pelvic discontinuity. The overall complication rate was 10.5% (2/19), comparable to international data; only one case was directly related to the acetabular component and required re-intervention. No deaths, dislocations, or mechanical implant failures were recorded.

A longer follow-up is required to assess implant fixation and the durability of the reconstruction.

## Figures and Tables

**Table 1:** Demographic and clinical characteristics of patients.

Parameter	Value
Patients, n	19
Sex: male/female, n	10/9
Age, years, mean ± SD (range)	53.6 ± 11.0 (39–72)
Side: left/right, n (%)	11 (57.9)/8 (42.1)
Number of previous surgeries, mean ± SD (range)	3.0 ± 2.1 (1–9)
Time from primary implant to revision, years, mean ± SD (range)	13.7 ± 6.2 (4–25)
History of periprosthetic infection, n (%)	8 (42.1)
Paprosky defect: IIIA/IIIB, n (%)	6 (31.6)/13 (68.4)
Pelvic discontinuity, n (%)	16 (84.2)

**Table 2:** Dynamics of functional outcomes (HHS, WOMAC, VAS) over 12 mo postoperatively (M ± SD).

Time Point	HHS	WOMAC	VAS
Preoperative	37.5 ± 5.2	74.5 ± 9.2	7.6 ± 1.0
3 mo	56.0 ± 6.3	59.9 ± 8.7	5.6 ± 0.9
6 mo	65.6 ± 7.8	50.2 ± 8.0	4.5 ± 0.8
12 mo	74.5 ± 8.6	40.3 ± 7.6	2.8 ± 0.7

Harris Hip Score (HHS); Western Ontario and McMaster Universities Osteoarthritis Index (WOMAC); Visual Analog Scale (VAS).

## Data Availability

The datasets supporting the findings of this study are available from the corresponding author upon reasonable request. The data are not publicly available due to ethical restrictions and patient confidentiality requirements.

## Abbreviations

3D—Three-dimensional; ASA—American Society of Anesthesiologists; CAD—Computer aided design; CDAMAC—Custom designed additive manufactured acetabular component; CI—Confidence interval; CT—Computed tomography; DICOM—Digital Imaging and Communications in Medicine; DMLS—Direct metal laser sintering; HHS—Harris Hip Score; ICM—International Consensus Meeting; MSCT—Multislice computed tomography; NJR—National Joint Registry (UK); PJI—Periprosthetic joint infection; SD—Standard deviation; STROBE—Strengthening the Reporting of Observational Studies in Epidemiology; THA—Total hip arthroplasty; VAS—Visual Analog Scale; WOMAC—Western Ontario and McMaster Universities Osteoarthritis Index.
